# Mortality and Burden of Disease Attributable to Cigarette Smoking in Qingdao, China

**DOI:** 10.3390/ijerph13090898

**Published:** 2016-09-09

**Authors:** Yani Wang, Fei Qi, Xiaorong Jia, Peng Lin, Hui Liu, Meiyun Geng, Yunning Liu, Shanpeng Li, Jibin Tan

**Affiliations:** 1 Medical College, Qingdao University, Qingdao 266071, China; yanihezhu@126.com; 2 Center for Disease Control and Prevention, Qingdao 266033, China; qdqff@163.com (F.Q.); jxiaorong1@126.com (X.J.); qingmu0532@126.com (P.L.); liuhui90@126.com (H.L.); osdt2012@hotmail.com (M.G.); 3 Chinese Center for Disease Control and Prevention, Beijing 100050, China; liuyunning0723@163.com

**Keywords:** smoking, mortality, smoking impact ratio, PAF

## Abstract

In China, smoking is the leading preventable cause of deaths by a disease. Estimating the disease burden attributable to smoking contributes to an evaluation of the adverse impact of smoking. To aid in policy change and implementation, this study estimated the population-attributable fractions (PAFs) of smoking, the all-cause mortality and the loss of life expectancy attributable to smoking in 2014 of Qingdao. PAFs were calculated using the smoking impact ratio (SIR) or current smoking rate (P) and relative risk (RR). We determined the smoking-attributable mortality by multiplying the smoking-attributable fraction by the total mortality. This study used the method of an abridged life table to calculate the loss of life expectancy caused by smoking. Smoking caused about 8635 deaths (6883 males, 1752 females), and accounted for 16% of all deaths; 22% in males and 8% in females. The leading causes of deaths attributable to smoking were lung cancer (38%), ischemic heart disease (19%) and chronic obstructive pulmonary disease (COPD, 12%). The PAF for all causes was 22%; 30% in males and 10% in females. Tobacco use may cause a reduction of about 2.01 years of the loss of life expectancy; 3 years in males and 0.87 years in females. The findings highlight the need for taking effective measures to prevent initiation and induce cessation.

## 1. Introduction

Tobacco use is one of the most important public health concerns. Smoking can cause various diseases such as cardiovascular diseases (CVD), respiratory diseases and cancers of the lungs and multiple other sites [1,2] and account for the loss of 57 million disability-adjusted life-years (DALYs), becoming one of the top 10 risk factors for mortality [3].

A paper published in the Lancet [4] indicated that the annual number of deaths increased from 8.6 million in 1990 to 9.1 million in 2013 and the deaths due to non-communicable diseases increased by 33.5% (19.4%–46.9%) from 5.9 million to 7.9 million. Cerebrovascular disease became the leading cause of death in China, followed by cardiovascular diseases, chronic obstructive pulmonary disease (COPD) and lung cancer. Cancer incidence and mortality rates for some cancers in the USA and European countries had steadily decreased over the last decades, whereas the incidence and mortality of certain cancers in China had been increasing at an alarming speed [5]. Similar to what had occurred in the United States, unhealthy lifestyles in China, including smoking and an unbalanced diet, together with frightening levels of pollution, had contributed to a marked increase in chronic diseases [6]. “China Cardiovascular Disease Report 2014” clearly indicated that smoking is an independent risk factor for acute coronary events and acute ischemic strokes, and 19.9% of acute coronary heart disease and 11.1% of ischemic strokes were attributed to smoking [7]. Smoking will cause about 20% of all adult male deaths in China during the 2010s which equates to approximately 1 million deaths (840,000 male, 130,000 female) [8]. It was estimated that 2 million smokers would die every year in China due to smoking-related diseases by 2020 [9]. The economic loss attributed to smoking of adults above 35 years old was 2237.24 billion CNY in China [10].

China consumed over a third of the world’s cigarettes [11]. Evidence from the Global Adult Tobacco Survey in 2010 showed that there were more than 300 million smokers in China and the current smoking rate of residents aged 15 years and above was 28.1%. The current smoking rates fell by 0.9% per year on average between 1996 and 2002, while the current smoking rate fell by only 0.1% between 2002 and 2010. Although the smoking rate in Qingdao was at a low level, the mortality of diseases attributable to smoking reached a high level. Currently, we are in the early stages of effectively addressing the tobacco threat and facing an immense public health challenge. This study aimed to estimate the population-attributable fractions (PAFs) of smoking, the all-cause mortality due to tobacco use and the loss of life expectancy attributable to smoking in Qingdao.

## 2. Methods

### 2.1. Data Sources

Smoking prevalence: Data from 4349 participants who completed the Global Adult Tobacco Survey (GATS) 2014 in Qingdao were used to acquire the prevalence of smoking, quitting smoke, and passive smoking in urban and rural areas. This survey was not related to smokeless/spitless tobacco products but rather manufactured cigarettes. Stratified multi-stage cluster sampling was used to select 5000 households in Qingdao and one eligible respondent aged 15 years and above was randomly selected in each household. A standardized questionnaire was administered to the participants through a face to face interview by electronic devices, using the Global Tobacco Surveillance System. Data were weighted and analyzed by SAS 9.3 (Cary, New York City, NY, USA) and SUDAAN 10.0.1 (RTI International, Research Triangle Park, NY, USA) complex survey data analysis program.

All subjects gave their informed consent for inclusion before they participated in the study. The study was conducted in accordance with the Declaration of Helsinki, and the protocol was approved by the Ethics Committee of Chinese Center for Disease Control and Prevention (Project identification code: 201333).

Population: The population was obtained from the Qingdao Municipal Bureau of Statistics. The population by sex and age was shown in Table 1.

All-cause mortality database: Medical institutions at all levels filled in the death certificate, then reported the death information to the Center for Disease Control and Prevention of Qingdao by the online report system. The false negative rate was about 5%.

It’s necessary to obtain the lung cancer mortality data of a reference population and research region for calculating the smoking impact ratio (SIR). The mortality of 28 diseases attributable to smoking in Qingdao was gained from a death reporting system in Centers for Disease Control and Prevention of Qingdao. Lung cancer mortality for the reference population was gained from the American Cancer Society’s Cancer Prevention Study Phase II (ACS-CPS II) [12].

The relative risks of diseases for smokers were obtained from the meta-analysis in a large number of worldwide prospective cohort studies and a cross-sectional study [13].

### 2.2. Measurement of Tobacco Exposure

#### 2.2.1. Direct Method: Current Smoking Rate (P)

Applicable diseases: tuberculosis, lower respiratory infection, ischemic heart disease, ischemic stroke, hemorrhagic stroke, hypertensive heart disease, atrial fibrillation, aortic aneurysm, peripheral vascular disease, other cardiovascular system disease, asthma, diabetes.

#### 2.2.2. Indirect Method: Smoking Impact Ratio (SIR)

Applicable diseases: esophagus cancer, stomach cancer, liver cancer, lung cancer, cervical cancer, colorectal cancer, oral cancer, nasopharynx cancer, pancreatic cancer, kidney cancer, bladder cancer, leukemia, COPD, pneumoconiosis, interstitial lung disease, other chronic respiratory diseases.

The accumulated hazards of smoking were decided by factors such as the age at which smoking began or stopped, duration of smoking, number of cigarettes smoked per day, whether the tobacco product was in the form of cigarettes, bidis or cigars, and smoking behavior such as degree of inhalation [14]. The indicator of the current smoking rate alone cannot reflect the accumulated hazards of smoking.

Peto et al. [12] regarded the level of lung cancer mortality compared with never-smokers as an indicator of the accumulated hazards of smoking in a population. The SIR is defined as population lung cancer mortality in excess of never-smokers, relative to excess lung cancer mortality for a known reference group of smokers (CPS II). The accumulated hazards of smoking in the Qingdao population was measured in the form of the SIR which, together with relative risk estimates, was used to calculate PAFs. The SIR was calculated for all four population groups as follows:

(1) $$\mathrm{SIR}=\frac{C_{\mathrm{LC}}-N_{\mathrm{LC}}}{{S^{*}}_{\mathrm{LC}}-{N^{*}}_{\mathrm{LC}}}$$

where C_LC_ is the lung cancer mortality rate in the study population, N_LC_ is the lung cancer mortality rate of never-smokers in the same population, and S*_LC_ and N*_LC_ are lung cancer mortality rates for smokers and never-smokers in a reference population.

Due to exposure to other lung cancer risk factors such as coal use for heating and cooking, lung cancer rates in non-smokers in China are higher than in the CPS-II population [15]. The formula above needs to be normalized as follows:

(2) $$\mathrm{SIR}=\frac{C_{\mathrm{LC}}-N_{\mathrm{LC}}}{{S^{*}}_{\mathrm{LC}}-{N^{*}}_{\mathrm{LC}}}\times \frac{{N^{*}}_{\mathrm{LC}}}{N_{\mathrm{LC}}}$$

SIR captures the accumulated hazards of smoking by converting the smokers who may have different smoking histories in the Qingdao population into equivalents of smokers in the CPS-II population where hazards for other diseases have been measured [12].

### 2.3. Estimating the Effects of Smoking on Disease Outcomes

The fraction of deaths attributable to smoking was estimated with the standard population-attributable fraction (PAF). The sex-age-specific current smoking rate (P) or SIR and relative risk (RR) were substituted into the PAF formula:

(3) $$\mathrm{PAF}=\frac{P\left(\mathrm{RR}-1\right)}{P\left(\mathrm{RR}-1\right)+1}$$

Or:

(4) $$\mathrm{PAF}=\frac{\mathrm{SIR}\left(\mathrm{RR}-1\right)}{\mathrm{SIR}\left(\mathrm{RR}-1\right)+1}$$

where P is the current smoking rate (for some diseases, P is substituted by SIR).

The current smoking rate was used to calculate PAF, which was appropriate for some diseases with a shorter time interval between the exposure and disease outcomes such as cardiovascular disease, tuberculosis and other respiratory diseases than lung cancer and chronic obstructive pulmonary disease. The study of Ezzati [15] indicated that the mortality of cardiovascular disease was reduced within two years after quitting smoking, but the mortality of lung cancer and chronic obstructive pulmonary disease was reduced within five to ten years after quitting. This means that the influence of a past smoking rate on lung cancer was greater than the influence on cardiovascular disease. Because the SIR is calculated by lung cancer mortality, the PAF calculated by SIR can give an index to the influence of current smoking and former smoking status on the mortality of lung cancer and other diseases which have a longer time interval between the exposure and disease outcomes.

### 2.4. Calculating the Sex-Age-Specific Disease Mortality

Mortality estimation procedures involved adjustments for under-reporting, corrections for age, sex and plausibility errors, and redistribution of ill-defined codes. Based on the data of the death reporting system in Qingdao, diseases from the death database were distributed to 253 categories of diseases to obtain the sex-age-specific disease mortality. Among them, there were 28 categories of diseases related to smoking.

### 2.5. Calculating the Influence of Smoking on Disease Death and Life Expectancy

#### 2.5.1. Measuring the Smoking-Attributable Mortality

The formula of attributable mortality (AM) is as follow:

(5) AM = PAF × M

where “M” denotes the sex-age-specific deaths of some diseases which have etiology contact with smoking and “PAF” denotes the standard population-attributable fraction.

#### 2.5.2. Calculating the Loss of Life Expectancy Attributed to Smoking

According to the level of mortality, an abridged life table was used to calculate the overall life expectancy and the life expectancy eliminating deaths due to smoking. The difference between these two life expectancies was the loss of life expectancy caused by smoking.

## 3. Results

### 3.1. The Numbers and Proportion of Deaths Attributable to Smoking

There were 54,048 deaths (30,860 in males and 23,188 in females) among 253 diseases in Qingdao. The number of deaths attributable to smoking by sex are summarized in Table 2. Smoking caused about 8635 (6883 males, 1752 females) deaths, accounted for 16% of all deaths, 22% in males and 8% in females. The leading causes of deaths attributable to smoking were lung cancer (38%), ischemic heart disease (19%) and COPD (12%). Among the 6883 deaths caused by smoking in males, lung cancer, ischemic heart disease and COPD deaths accounted for 65%. Among 1752 deaths caused by smoking in females, the deaths of these three diseases accounted for 82%.

### 3.2. PAFs by Sex and Age

PAF by sex are shown in Table 2. The PAF for cancer, respiratory diseases and cardiovascular disease were 30%, 43% and 14% respectively. The PAF for all-cause mortality was 22% (30% in males and 10% in females). In males, lung cancer showed the greatest PAF (68%), followed by COPD (48%) and nasopharynx cancer (45%), while in females COPD showed the greatest PAF (52%), followed by lung cancer (51%) and oral cancer (30%).

The number of deaths attributable to smoking and PAF of diseases by age and sex are shown in Table 3. There were increasing trends in deaths attributable to smoking with age. PAFs were higher in men than women for all age categories.

### 3.3. Life Expectancy and Life Expectancy Eliminating the Influence of Smoking

The life expectancy in Qingdao was 80.93; 78.18 in males and 83.89 in females. If smoking is eliminated, the life expectancy will increase by 2.01 years; 3 years in males and 0.87 years in females.

## 4. Discussion

### 4.1. The Leading Cause of Deaths Attributable to Smoking

Lung cancer became the leading cause of deaths attributable to smoking in Qingdao, which was similar to the nationwide cause [16] but different to the global burden of disease study [17] that found cardiovascular disease to make up a much larger proportion of total smoking-attributable deaths. In China, compared with 1990, the disease burden of lung cancer in 2010 had a risen greatly [18].

### 4.2. The Diseases Which Have Close Association with Smoking

In males, tobacco smoking was responsible for 68% of lung cancer deaths, 48% of COPD, 40%–45% of other cancers (including nasopharynx cancer and oral cancer). In females, smoking was responsible for around 50% of lung cancer and 50% of COPD deaths. Thus it can be seen that lung cancer, COPD, nasopharynx cancer and oral cancer had a close association with smoking.

We estimate that, including only the 11 established smoking-attributable cancers, the overall PAF was 30%. The PAF was somewhat higher in men (34%) than in women (23%). These results were similar to the PAFs in the United States [19]. Undoubtedly, declines in smoking prevalence contributed to the decline of both the rate of cancer mortality attributable to smoking and the PAF. However, the PAF for smoking and cancer mortality estimated in this analysis is similar to the 30% estimated by Doll and Peto [20] more than 30 years ago. Some factors contributed to increasing the PAF estimate, including increases over time in the RR of lung cancer mortality among smokers [21], and decreases in cancer mortality for reasons unrelated to smoking.

### 4.3. The Proportion of Deaths Attributable to Smoking

Smoking caused about 26.32% of all male deaths at ages 40–79 years, but 20% in the nationwide prospective cohort studies during the 2010s [8]. Furthermore, smoking caused about 10.41% of all female deaths at ages 40–79 years, which was higher than the nationwide percentage (3%). There was a high current smoking rate at ages 40–79 years in Qingdao. Moreover, the epidemiological studies showed that Qingdao had a high incidence of lung cancer; the lung cancer death rate was far higher than the national average death rate and was also higher than the average death rate of major cities in China [22]. Smoking was not only harmful to human health, but also caused environmental pollution, which was bad for acute cardiovascular and cerebrovascular disease.

### 4.4. Challenges to the Smoking Control

In China, 52.1% of males smoke, more than half of whom say they have never tried to quit. A total of 80% of Chinese people are aware that smoking would cause lung cancer, but only 42.6% of Chinese people were aware of the bad influence of smoking on strokes and myocardial infarction [23]. In the past 15 years, the proportion of deaths attributable to smoking had increased to double at ages 35–74 years [8]. In 2011, locally regulated guidelines were issued to ban smoking in indoor public places, and signs prohibiting smoking are common in restaurants and bars. However, poor enforcement and few penalties for non-compliance mean that the rule is widely ignored [24]. In 2013, the regulation of tobacco control in Qingdao was issued to ban smoking in indoor public places, indoor workplaces and public transport. This regulation contributed to reducing the smoking rate in Qingdao. Although the smoking prevalence rate of Qingdao was at a low level, the burden of diseases attributable to smoking was relatively heavy. The adverse effects of smoking sometimes emerged after a long time. So the current states of death attributable to smoking reflected the previous smoking prevalence level. Currently, a low smoking rate may lead to a low mortality rate, attributable to smoking in the future. If smoking is eliminated, the life expectancy will increase by 2.01 years in Qingdao. It is urgent to take effective measures to accelerate cessation and to halt and reverse the status of increasing deaths attributable to smoking.

### 4.5. Advantages

This study was the first attempt at estimating the burden of diseases attributable to smoking in Qingdao where the smoking prevalence level was at a very low level. In contrast, to not just rely on the method of calculating PAFs by current smoking rate, this study also used SIR to calculate the PAF of some diseases that had a longer time interval between the exposure and disease outcomes. In order to obtain a more precise prevalence rate of smoking, a large-scale adult tobacco survey had been carried out in Qingdao. Moreover, the RR of current smokers versus non-smokers was provided by recent published articles with comprehensive data. Therefore the estimation of PAFs was more precise.

### 4.6. Limitations

First, this study may underestimate deaths attributable to smoking because the former smokers were not taken into account. We were not able to estimate the deaths related to pipe smoking and smokeless tobacco use due to the data availability. Second, due to the absence of a smoking prevalence rate in the past few years, comparisons of the burden of diseases attributable to smoking cannot be made with previous results in Qingdao. Third, the sample size of this study is relatively small and can only reflect the status of Qingdao.

### 4.7. Further Research

In order to compare the burden of diseases related to smoking between different years, large-scale studies of tobacco use should be conducted in the future, taking former smokers, pipe smokers and smokeless tobacco users into account.

## 5. Conclusions

In 2014, smoking caused about 8635 deaths due to smoking, and accounted for 16% of the total deaths in Qingdao. The overall PAF estimate of smoking was 22%. To prevent initiation or to induce cessation, it is recommended that more rigorously implement policy measures should be carried out in Qingdao.

## Figures and Tables

**Table 1:** The population of Qingdao by sex and age.

**Gender**	**0–**	**1–**	**5–**	**10–**	**15–**	**20–**	**25–**	**30–**	**35–**	**40–**
male	42,253	147,487	191,001	180,315	193,817	195,101	300,471	293,484	279,038	355,969
female	38,686	134,076	177,921	170,759	186,721	188,188	306,037	308,070	287,691	357,914
total	80,939	281,563	368,922	351,074	380,538	383,289	606,508	601,554	566,729	713,883
**Gender**	**45–**	**50–**	**55–**	**60–**	**65–**	**70–**	**75–**	**80–**	**85–**	
male	351,984	319,328	294,574	262,027	165,978	114,831	95,304	62,030	37,569	
female	358,931	320,460	292,785	267,342	166,734	114,189	104,384	79,179	63,710	
total	710,915	639,788	587,359	529,369	332,712	229,020	199,688	141,209	101,279	

**Table 2:** Number of deaths attributable to smoking and population-attributable fractions (PAF) of diseases by sex.

Diseases	Deaths Attributable to Smoking			Deaths of Diseases			PAF (%)		
	Men	Women	Total	Men	Women	Total	Men	Women	Total
Cancer	3251	1123	4374	9632	4956	14,588	34	23	30
Esophagus cancer	287	27	314	760	92	852	38	30	37
Stomach cancer	155	37	192	1782	799	2581	9	5	7
Liver cancer	343	45	388	2254	789	3043	15	6	13
Lung cancer	2310	942	3252	3398	1834	5232	68	51	62
Cervical cancer		16	16		235	235		7	7
Colorectal cancer	20	13	33	631	370	1001	3	4	3
Oral cancer	17	4	21	41	13	54	42	30	39
Nasopharynx cancer	17	13	30	38	45	83	45	29	37
pancreatic cancer	47	14	60	330	165	495	14	8	12
Kidney cancer	9	2	11	72	44	116	12	4	9
Bladder cancer	30	4	34	172	39	211	17	11	16
Leukemia	15	7	22	154	531	685	10	1	3
Respiratory diseases	720	358	1078	1640	856	2496	44	42	43
Chronic obstructive pulmonary disease (COPD)	656	348	1004	1360	669	2029	48	52	49
Pneumoconiosis	1	1	2	13	8	21	10	7	9
Interstitial lung disease	7	3	10	75	43	118	9	7	8
Other chronic respiratory diseases	0	0	1	2	4	6	9	8	8
Tuberculosis	8	0	8	44	13	57	19	2	15
lower respiratory infection	18	2	20	44	43	87	41	5	23
Asthma	29	4	33	102	76	178	28	5	18
Cardiovascular disease	2856	267	3123	11,264	10,424	21,688	25	3	14
Ischemic heart disease	1499	147	1646	5942	5718	11,660	25	3	14
Ischemic stroke	584	65	648	2607	2470	5077	22	3	13
Hemorrhagic stroke	650	46	695	2211	1737	3948	29	3	18
Hypertensive heart disease	89	8	98	393	457	850	23	2	12
Atrial fibrillation	0	0	0	0	2	2		2	2
Aortic aneurysm	19	0	20	58	22	80	34	2	25
Peripheral vascular disease	1	0	1	3	0	3	44		44
Other cardiovascular system disease	13	0	14	50	18	68	27	2	20
Others									
Diabetes	57	4	61	412	655	1067	14	1	6
All causes	6883	1752	8635	22,948	16,891	39,839	30	10	22

**Table 3:** Number of deaths attributable to smoking and PAF of diseases by age and sex.

Age Group (Years)	Current Smoking Rate (%)			Deaths Attributable to Smoking			Deaths of Diseases			PAF (%)		
	Men	Women	Total	Men	Women	Total	Men	Women	Total	Men	Women	Total
30–34	36.02	0.64	18.45	28	7	35	82	51	133	35	14	27
35–39	39.56	0	22.52	57	19	76	149	78	227	38	24	33
40–44	48.65	0.99	22.73	153	36	189	418	172	590	37	21	32
45–49	58.02	0.93	29.42	367	63	430	774	315	1089	47	20	40
50–54	49.10	0	24.95	440	63	503	1091	398	1489	40	16	34
55–59	58.56	0.21	29.56	758	118	876	1812	663	2475	42	18	35
60–64	51.45	2.46	26.75	863	147	1010	2334	984	3318	37	15	30
65–69	47.16	0.44	26.35	788	138	926	2276	1092	3368	35	13	27
70–74	36.33	4.32	22.23	842	198	1040	2719	1462	4181	31	14	25
75–79	33.48	3.04	19.79	998	322	1320	3695	2512	6207	27	13	21
80–84	27.25	4.05	16.10	835	396	1231	3784	3530	7314	22	11	17
85+	26.71	2.28	16.61	753	246	999	3814	5634	9448	20	4	11
Total	40.53	1.04	21.31	6883	1752	8635	22,948	16,891	39,839	30	10	22
